# Administrative integration of vertical HIV monitoring and evaluation into health systems: a case study from South Africa

**DOI:** 10.3402/gha.v6i0.19252

**Published:** 2013-01-24

**Authors:** Mary Kawonga, Sharon Fonn, Duane Blaauw

**Affiliations:** 1 Gauteng Health Department, Charlotte Maxeke Johannesburg Academic Hospital, Johannesburg, South Africa; 2 School of Public Health, Faculty of Health Sciences, University of the Witwatersrand, Johannesburg, South Africa; 3 Centre for Health Policy, School of Public Health, Faculty of Health Sciences, University of the Witwatersrand, Johannesburg, South Africa

**Keywords:** integration, vertical programme, disease-specific intervention, health system strengthening, monitoring and evaluation, district health system, South Africa

## Abstract

**Background:**

In light of an increasing global focus on health system strengthening and integration of vertical programmes within health systems, methods and tools are required to examine whether general health service managers exercise administrative authority over vertical programmes.

**Objective:**

To measure the extent to which general health service (horizontal) managers, exercise authority over the HIV programme's monitoring and evaluation (M&E) function, and to explore factors that may influence this exercise of authority.

**Methods:**

This cross-sectional survey involved interviews with 51 managers. We drew ideas from the concept of ‘exercised decision-space’ – traditionally used to measure local level managers’ exercise of authority over health system functions following decentralisation. Our main outcome measure was the degree of exercised authority – classified as ‘low’, ‘medium’ or ‘high’ – over four M&E domains (HIV data collection, collation, analysis, and use). We applied ordinal logistic regression to assess whether actor type (horizontal or vertical) was predictive of a higher degree of exercised authority, independent of management capacity (training and experience), and M&E knowledge.

**Results:**

Relative to vertical managers, horizontal managers had lower HIV M&E knowledge, were more likely to exercise a higher degree of authority over HIV data collation (OR 7.26; CI: 1.9, 27.4), and less likely to do so over HIV data use (OR 0.19; CI: 0.05, 0.84). A higher HIV M&E knowledge score was predictive of a higher exercised authority over HIV data use (OR 1.22; CI: 0.99, 1.49). There was no association between management capacity and degree of authority.

**Conclusions:**

This study demonstrates a HIV M&E model that is neither fully vertical nor integrated. The HIV M&E is characterised by horizontal managers producing HIV information while vertical managers use it. This may undermine policies to strengthen integrated health system planning and management under the leadership of horizontal managers.

Efforts to improve health in low- and middle-income countries (LMIC) are often characterised by tensions between horizontal approaches, which seek to tackle health problems ‘on a wide front, through the creation of a system of permanent institutions commonly known as general health services’ (1), and vertical approaches, which tackle one specific health problem through targeted delivery, coordination, financing, or information mechanisms (2, 3).While vertical approaches increase the coverage of targeted interventions, their parallel mechanisms undermine and fragment health systems (4). For example, a vertical approach to the scale-up of antiretroviral treatment (ART) attracts staff away from general services and establishes parallel drug supply mechanisms that bypass and undermine national systems, whereas a horizontal approach strengthens capacity of general services and existing health system drug-delivery systems which include catering for ART scale-up needs (5). Thus, approaches that maximise synergies between health systems and programmes are recommended, including the diagonal approach to strengthen health systems through pursuing specific disease priorities (6, 7), or full integration of vertical programmes within health systems (8).

Integration is most commonly described as providing two or more vertical services at the same point of care (9–11). Increasingly it is understood as integrating policies, management (administrative integration) or implementation activities (operational integration) of vertical programmes within health system functions such as governance, service delivery, financing, or monitoring and evaluation (M&E) (12, 13). Data relating to policy and operational integration are becoming more available (12, 14, 15), but less so for administrative integration (16). Unger et al. (13) conceptualise administrative integration as: integrating the middle management of disease-specific programmes within general service management, giving general service (horizontal) managers administrative authority over disease-specific activities, and disease-specific (vertical) managers providing technical advice. This implies a shift in day-to-day administrative responsibility over disease-specific interventions from vertical to horizontal managers. For example, for the health system M&E function (which entails data collection, collation, analysis, and use (17)), administrative integration might mean that horizontal managers exercise authority in coordinating collection and collation of disease-specific data and refer to vertical managers for technical advice on how to use these data for management. Whether this model of administrative integration is happening in South Africa has not been documented.

## Vertical programmes and the health system in South Africa

In South Africa, integration is a health sector reform priority, while several vertical programmes exist, notably for HIV, tuberculosis (TB), and maternal and child health (MCH) (18). For example, the HIV programme was introduced soon after 1994 with earmarked funding (19) and later a conditional grant (20), as well as a dedicated M&E system (21). Initially focussed on prevention, it has evolved over time to include ART; HIV services have been progressively integrated within general health services (22, 23). However, historically a national HIV/AIDS directorate and HIV managers at provincial and district levels have controlled the programme. Furthermore, HIV programme managers account for the HIV conditional grant by submitting data and financial reports to the National Treasury through dedicated reporting mechanisms. Also, several sub-programmes (e.g. for HIV counselling and testing [HCT], prevention of mother-to-child HIV transmission [PMTCT], and ART) have been established within the HIV programme and are coordinated in separate silos, indicating further verticalisation (24). This is at odds with current health sector decentralisation reforms that envisage integrated health management under the control of horizontal managers at district level. South Africa's decentralisation reforms entail: the devolution of political and administrative authority from national level to nine semi-autonomous provincial governments (25), and the establishment of a district health system (DHS) by shifting health management responsibility from provincial to district health clusters (deconcentration) (26, 27). A nationally standardised district health information system (DHIS) has been established to support DHS management (28).

If the DHS is to be the foundation of the health system as envisaged, then district managers need to exercise authority over DHS functions, including disease-specific interventions (i.e. administrative integration). This study examines whether this is happening. We use the HIV programme as a case study given its traditionally vertical approach, and focus on the M&E (information) function as a tracer for analysing administrative integration (24). Our study therefore aims to: describe the extent to which horizontal managers exercise authority over HIV M&E coordination, determine factors associated with exercised authority, and explore vertical managers’ roles in HIV M&E coordination. We hypothesise that vertical managers and those with higher management capacity and HIV M&E knowledge exercise more authority.

## Method

### Study design and setting

We conducted a cross-sectional study during 2010–2011 in two of South Africa's nine provinces (one rural [Site A], one urban [Site B]). Each province comprises several districts, each with sub-districts further divided into local areas (clusters of health facilities). In both sites, horizontal managers at district, sub-district, local area, and facility levels coordinated general health services. Both HIV programmes were coordinated by a senior HIV manager, 3–4 sub-programme HIV managers and several assistant sub-programme managers at provincial level, assisted by one HIV manager each at district, sub-district (Site A only), and district hospital levels. Horizontal information managers coordinated the DHIS. At the time of this study, HIV data recording and reporting were integrated within the DHIS in Site B (though HIV data were recorded by dedicated staff and coordinated by a HIV M&E manager). In Site A, HIV prevention data were fully integrated within the DHIS, while ART data were coordinated separately by a HIV M&E manager (24).

### Study population and sampling

**Fig. 1:**
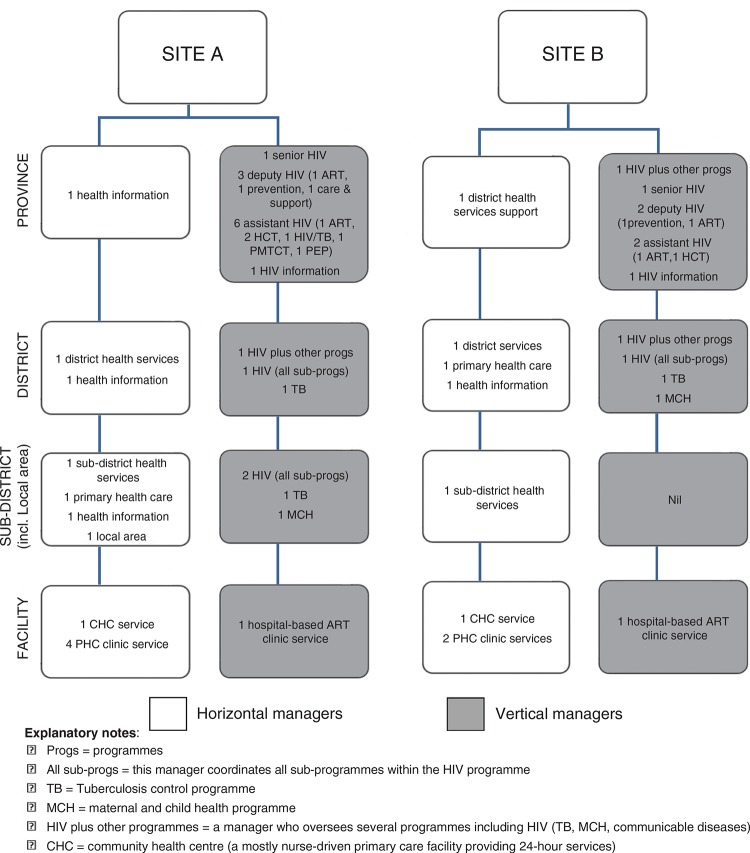
Study participants at each level of the health system.

We purposively selected sites where we have on-going research. We included only public sector services. We selected one of three districts in Site A, and one of six in Site B, and within each district selected one sub-district and one local area per sub-district. All primary care facilities within a local area, as well as the district hospital HIV service were included (representing the full spectrum of HIV services: those initiating patients on ART; those to which patients started on ART who are referred for on-going care; and those providing HIV prevention only [HCT, PMTCT, and HIV/TB]). Our inclusion criteria were: i) manager works at any level from facility to provincial; ii) manager is primarily responsible for general health services or information (horizontal manager) or for vertical services or information (vertical manager); iii) manager coordinates the production of HIV information (data collection, collation, analysis) and/or uses HIV information. Of 53 managers selected, 51 participated (Fig. 1).

### Approach for measuring ‘exercised authority’

There are no existing tools to measure ‘exercised authority’ over programme administration. Bossert's ‘decision-space’ approach however provides a useful frame (29). Bossert's tool collects data to rate the degree to which sub-national officials exercise decision-making authority over health system functions after decentralisation as ‘narrow’, ‘moderate’ or ‘wide’. As it has never been applied to either the health system M&E function or to other programmes, we could not use it as is. However, we followed a similar approach to measure ‘exercised (administrative) authority’.

**Fig. 2:**
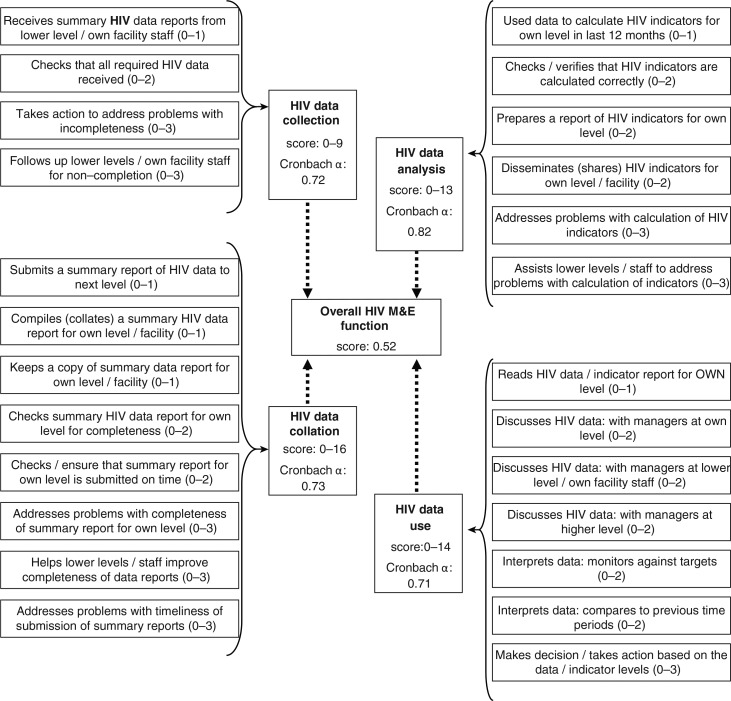
Sub-scales and items for measuring the extent to which managers performed HIV programme M&E tasks.

We defined exercised authority over the HIV M&E function as: *a manager undertakes tasks to oversee HIV data collection, collation and analysis, and uses HIV data to review the programme and take action*. To measure this, we first identified the M&E tasks that managers are reasonably expected to perform within each M&E domain (collection, collation, analysis, use) by consulting the M&E literature and using information from an expert informant (senior provincial manager). We then defined which were ‘routine administration’, ‘problem identification’, or ‘problem solving’ tasks (Figure 2). Finally, we designed, pilot-tested, and administered a semi-structured questionnaire to collect data on participants’ performance of these tasks. Our face-to-face interviews also collected data on: participant characteristics (age, sex, duration in current job, health system level); management capacity (training in human resource, financing, and health information management; duration of management experience); and M&E knowledge (defines common HIV indicators, differentiates counts and proportions, and understands the utility of three HIV indicators listed on the DHIS). Ethical approval was obtained from the University of the Witwatersrand Committee for Research on Human Subjects and both Provincial Health Departments. Participants gave written informed consent. Interviews were recorded and transcribed if separate consent to record was granted. Otherwise, detailed notes were taken and later typed in full.

We developed four sub-scales to measure the degree of exercised authority for each M&E domain. Sub-scales comprised several items (M&E tasks), which we coded ‘no’ if a respondent did not perform the task (score=zero) or ‘yes’ if s/he did. Tasks were weighted relative to their importance, so ‘yes’ responses were scored one for ‘routine administration’, two for ‘problem identification’ or three for ‘problem solving’ tasks. Item scores summed to a sub-scale score, and sub-scale scores summed to an overall HIV M&E score (Fig. 2). Cronbach's α coefficients for sub-scales ranged from 0.7 to 0.8. Scale structure was confirmed by principal component analysis (PCA). Extracted components were very similar to our sub-scales, so we used our sub-scales in the analysis. We also developed two composite scores for management capacity (7 items, score 0–13) and M&E knowledge (7 items, score 0–13).

### Data analysis

Data were analysed in SPSS 20.0 for Windows. We performed *t*-tests, Chi-square, and Mann–Whitney U tests to compare participant characteristics between the two sites, and mean scale and sub-scale scores between vertical and horizontal managers (alpha level of 0.05 for all tests). To determine the degree of exercised authority, we computed an ordinal dependent variable for each HIV M&E domain by categorising the maximum total score for each sub-scale into thirds. We then coded observed scores in the bottom, middle, and top third as ‘low’, ‘medium’, and ‘high’, respectively. We compared the distribution of dependent variables between horizontal and vertical managers (Chi-square test). We also performed ordinal logistic regression to investigate which explanatory variables (actor type [horizontal vs. vertical], health system level, study site, highest qualification, duration of management experience, management capacity score, and M&E knowledge score) were predictive of higher degrees of exercised authority. Finally, to determine vertical managers’ roles in HIV M&E coordination, we thematically analysed our narrative data, and also determined the proportion who undertook ‘routine administration’ versus ‘problem-solving’ tasks.

## Results

More than 75% of participants were female, with an undergraduate degree or higher, and had some management training. Site B managers had higher educational qualifications (Table 1). Participant characteristics were similar between vertical and horizontal managers (data not shown), except fewer horizontal managers had a postgraduate qualification (5% vs. 23%, *p=*0.02) and fewer were at provincial level (10% vs. 58%, *p=*0.02). Table 2 shows that horizontal managers attained higher mean scores for HIV data collection (*p*<0.05) and collation (*p*<0.01), but lower for HIV data use (*p<* 0.01) and M&E knowledge (*p<*0.05).

**Table 1:** Participant demographic and professional characteristics

Variable	All (*n=*51)	Site A (*n*=31)	Site B (*n*=20)	Significance
Age (years)+				
Mean (SD)	48.5 (7.5)	47 (6.9)	51 (7.9)	0.060
Sex				
Female# No. (%)	42 (82.4)	23 (74.2)	19 (95.0)	0.057
Disciplinary background# No. (%)				
Nursing	41 (80.4)	24 (77.4)	17 (85.0)	0.239
Non-health	7 (13.7)	6 (19.4)	1 (5.0)	
Medical	3 (5.9)	1 (3.2)	2 (10.0)	
Actor type# No. (%)				
General manager	20 (39.2)	12 (38.7)	8 (40.0)	0.927
Programme manager	31 (60.8)	19 (61.3)	12 (60.0)	
Level of health system# No. (%)				
Facility	10 (19.6)	6 (19.4)	4 (20.0)	0.185
Sub-district (includes local area)	9 (17.6)	8 (25.8)	1 (5.0)	
District	12 (23.5)	5 (16.1)	7 (35.0)	
Province	20 (39.2)	12 (38.7)	8 (40.0)	
Highest qualification attained# No. (%)				
Undergraduate diploma	9 (17.6)	4 (12.9)	5 (25.0)	0.007**
Undergraduate degree	31 (60.8)	24 (77.4)	7 (35.0)	
Postgraduate qualification	11 (21.6)	3 (9.7)	8 (40.0)	
Whether had management training# No. (%)				
Human resources	38 (74.5)	20 (64.5)	18 (90.0)	0.041*
Financial management	45 (88.2)	25 (80.6)	20 (100.0)	0.036*
Information management	43 (84.3)	25 (80.6)	18 (90.0)	0.370
Duration in current job@ (months)				
Median (IQR)	56 (22–66)	38 (60–148)	60 (24–66)	0.389
Management experience to date@ (months)				
Median (IQR)	82 (60–139)	95 (60–139)	73 (60–148)	0.685

+ *t*-test

# Pearson Chi-square test

@ Mann–Whitney U test.

* *p*<0.05.

** *p*<0.01.

**Table 2:** Capacity, M&E knowledge and HIV M&E scale and sub-scale scores

Scales and sub-scales	All managers (*n*=51)	Horizontal managers (*n*=20)	Vertical managers (*n*=31)	Significance#
Data collection (sub-scale score: 0–9)				
Mean (SD)	5.24 (3.2)	6.50 (2.6)	4.42 (3.4)	0.016*
Mean score as % of maximum possible score	58.2	72.2	49.1	
Data collation (sub-scale score: 0–16)				
Mean (SD)	8.24 (4.2)	10.10 (3.0)	7.03 (4.4)	0.005**
Mean score as % of maximum possible score	51.5	63.1	43.9	
Data analysis (sub-scale score: 0–13)				
Mean (SD)	3.43 (3.6)	4.60 (1.0)	2.68 (0.5)	0.098
Mean score as % of maximum possible score	26.4	35.4	20.6	
Data use (sub-scale score: 0–14)				
Mean (SD)	8.59 (3.12)	6.75 (5–10)	9.77 (9–12)	0.002**
Mean score as % of maximum possible score	61.4	48.2	69.8	
Overall M&E function (scale score: 0–52)				
Mean (SD)	25.49 (8.8)	27.95 (8.6)	23.90 (8.6)	0.108
Mean score as % of maximum possible score	49.0	53.8	46.0	
Management capacity (scale score: 0–13)				
Mean (SD)	6.12 (2.4)	5.85 (2.0)	6.29 (2.7)	0.534
Mean score as % of maximum possible score	47.1	45.0	48.4	
HIV M&E knowledge (scale score: 0–13)	(*n*=47)	(*n*=19)	(*n*=28)	
Mean (SD)	8.11 (3.4)	6.58 (3.2)	9.14 (3.3)	0.011*
Mean score as % of maximum possible score	62.4	50.6	70.3	

SD, standard deviation.

# Two-sided *t*-test.

* *p*<0.05.

** *p*<0.01.

### Degree of exercised authority

**Fig. 3:**
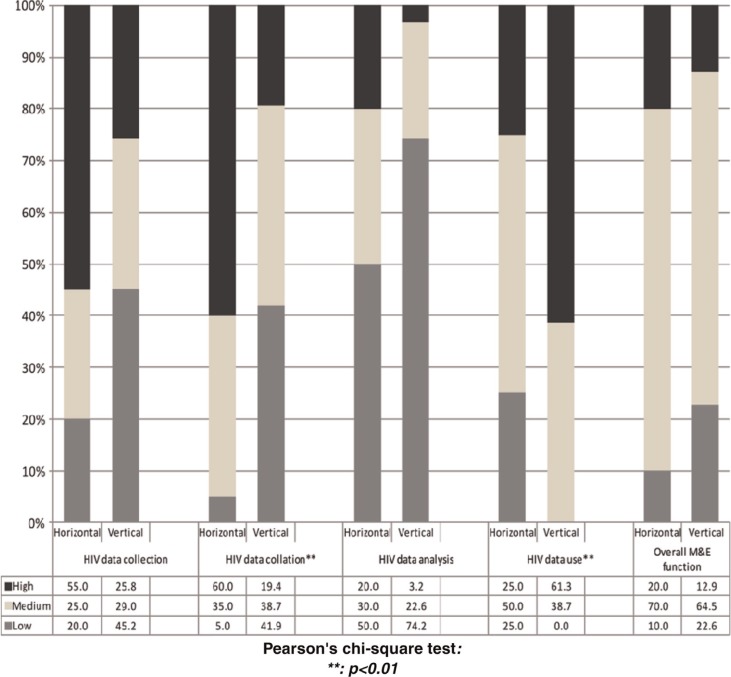
Degree of exercised authority: comparing distributions between horizontal and vertical managers.

A pooled analysis showed that 65% of all managers exercised a ‘medium’ degree of authority on the overall HIV M&E function, with no significant differences between vertical and horizontal managers. Disaggregating the data into collection, collation, analysis and use revealed differences between horizontal and vertical managers: more horizontal managers (60% vs. 19%; *p*=0.003) exercised a high degree of authority over HIV data collation, but fewer exercised a high degree over HIV data use (25% vs. 61%; *p*=0.003) (Fig. 3). Since four of the six information managers (whose primary role is to coordinate data) were horizontal managers, we performed Chi-square analysis excluding information managers. However, differences between horizontal and vertical managers persisted for HIV data collation (50% vs. 14%; *p*=0.001) and data use (31% vs. 71%, *p=*0.011).

We performed ordinal logistic regression only for the HIV data collation and data use domains (where Chi-square analyses showed associations). As Table 3 shows, horizontal managers, facility managers, and managers in Site A exercised higher degrees of authority over HIV data collation, while being a vertical manager, working at provincial level, having a postgraduate qualification or a higher M&E knowledge score were associated with exercising a higher degree of authority over HIV data use. Individual management capacity was not associated with exercised authority for either domain. Multivariate logistic regression revealed that being a horizontal manager was predictive of higher degrees of authority over HIV data collation (OR 7.26; CI: 1.9, 27.4). Being a vertical manager (OR 0.19; CI: 0.05, 0.84) and having a higher HIV M&E knowledge score (OR 1.22; CI: 0.99, 1.49) were predictive of higher degrees of authority over HIV data use (Table 3).

**Table 3:** Predictors of higher degrees of exercised authority: HIV data collation and data use

	HIV data collation		HIV data use	
		
Variable	Crude OR (95% CI)	Adjusted OR (95% CI)	Crude OR (95% CI)	Adjusted OR (95% CI)
Actor type				
Horizontal manager	7.5 (2.3, 24.8)	7.26 (1.9, 27.4)	0.15 (0.04, 0.51)	0.19 (0.05, 0.84)
Vertical manager	1		1	1
Study site				
Site A	2.0 (1.7, 5.6)	2.23 (0.63, 7.94)	0.86 (0.29, 2.52)	–
Site B	1	1	1	
Level of health system#				
Facility	7.9 (1.6, 40.3)	–	0.10 (0.02, 0.53)	–
Sub-district	1.3 (0.3, 5.5)		0.26 (0.05, 1.25)	
District	1.0 (0.3, 3.9)		0.72 (0.17, 3.07)	
Province	1		1	
Highest qualification				
Undergraduate diploma	3.1 (0.6, 16.7)	2.28 (0.4, 13.1)	0.35 (0.05, 2.17)	1.02 (0.12, 8.42)
Undergraduate degree	6.3 (1.6, 25.3)	2.44 (0.5, 12.5)	0.21 (0.05, 0.93)	0.57 (0.10, 3.38)
Postgraduate qualification	1	1	1	1
Management experience (duration in months)	1.0 (1.0, 1.0)	–	1.00 (0.99, 1.01)	–
Management capacity score	0.9 (0.7, 1.1)	–	1.12 (0.89, 1.40)	–
M&E knowledge score	1.0 (0.9, 1.2)	–	1.26 (1.06, 1.51)	1.22 (0.99, 1.49)

# ‘Level of health system’ not included in multivariate analyses due to its high correlation with ‘actor type’.

OR, odds ratio; CI, confidence interval.

### Vertical managers’ roles in HIV M&E coordination

Vertical managers generally coordinated data that was specific only to their respective programme (HIV, MCH, TB) or HIV sub-programme (PMTCT, ART, HCT, TB/HIV). There were some overlaps, for example, MCH managers as well as PMTCT sub-programme managers coordinated PMTCT data, with no clarity about who was ultimately accountable. HIV data were largely used collectively in groups, for example, during district meetings attended by horizontal managers and district-based vertical managers (who formally reported to the head of the district health department). Provincial HIV managers belonged to separate management structures and discussed HIV data during HIV programme meetings (often including district-based HIV managers but excluding district horizontal managers). Even in site B where all HIV data were operationally integrated in the DHIS, HIV managers coordinated and used sub-sets of HIV data in their separate sub-programmes, exclusive of horizontal managers.

**Fig. 4:**
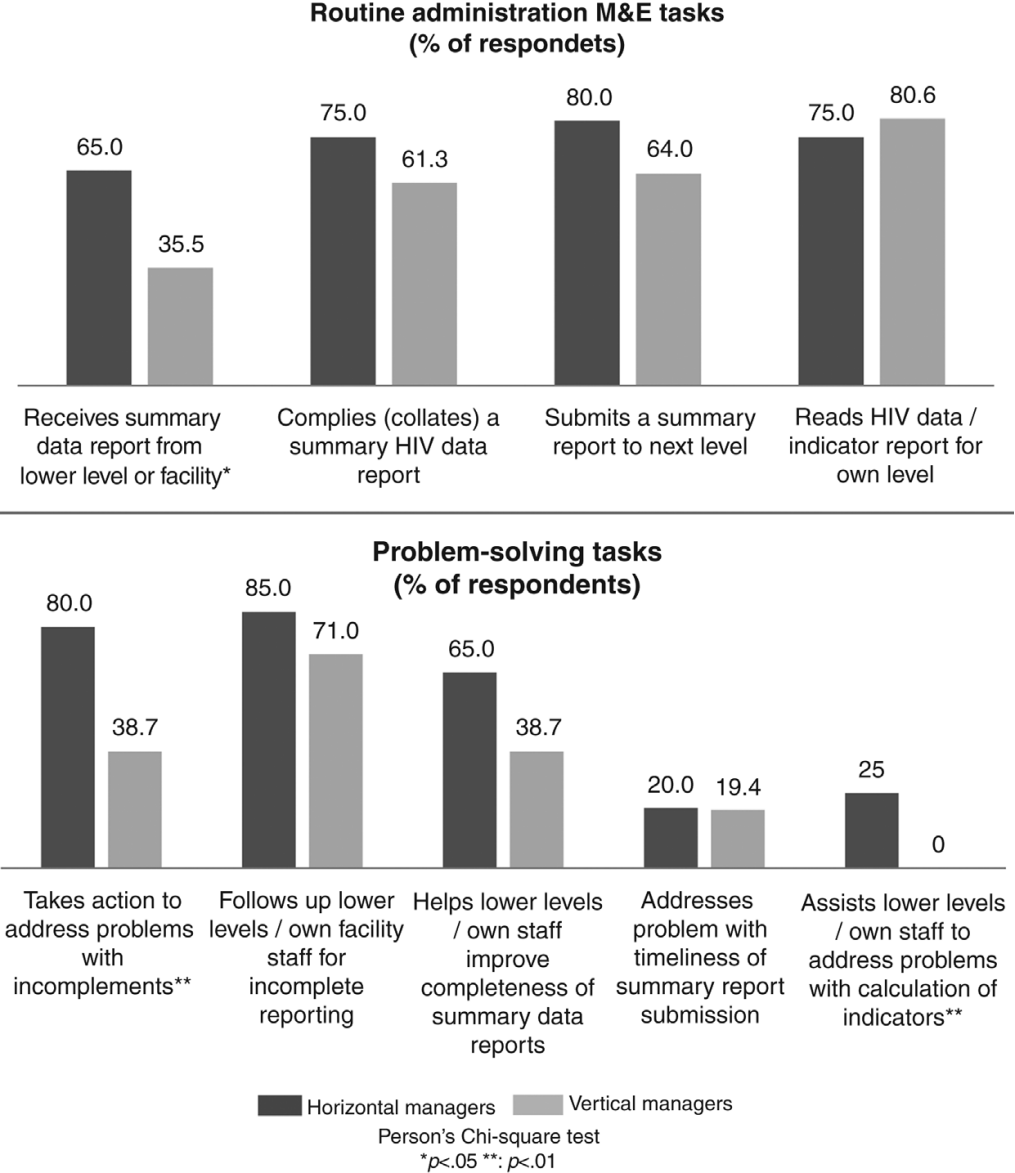
Managers’ roles in HIV M&E coordination.

Senior provincial HIV managers reportedly relied on sub-programme managers and district-based HIV managers for day-to-day HIV M&E coordination. Our quantitative data showed few provincial HIV managers playing a technical support role. Only a third or less, for example, helped horizontal managers at district or lower levels to address HIV M&E problems (Fig. 4).

## Discussion

This study adapted and applied an existing methodology to explore the measurement of administrative integration for the first time. Our discussion first addresses the application of decision space analysis in this study and related strengths and weaknesses. We then discuss our findings, taking into account the limitations of the study, and the implications of our findings for DHS strengthening. Based on this, we propose some recommendations.

### Applying decision space analysis

Given that this technique has never been used before in the way it was applied in this study, we must consider the validity of our measurements. We observed some unexpected findings, that is, general managers exercise greater authority than programme managers and management capacity was not associated with exercised authority. These could signify problems with our: a) measures or b) hypothesis (30). Regarding the latter, we formulated hypotheses premised on the assumption that the programme was completely vertically managed. However, the HIV M&E turned out to be a ‘hybrid’ model, and this may explain the unexpected observation. Regarding the former, two important considerations to note are: whether our scales comprehensively reflect the variables of interest (content validity), and the degree to which our tool measures the concept of exercised authority in relation to existing ideas (construct validity). For measuring ‘exercised authority’: in the absence of previously researched tools and formal delegation rules, we optimised content validity by devising sub-scales that we deemed comprehensively described HIV M&E domains, based on our knowledge of the M&E literature, consultation with an expert informant and pilot-testing. However, to measure management capacity, we only assessed whether participants had received training. Assessing the nature of this training may have revealed different results. The lack of previous similar studies limited our ability to test our scoring against a ‘gold standard’. However, having confirmed our scale structure with PCA, we are quite confident that our sub-scales are fairly robust.

Other potential limitations need to be considered. This technique requires that we describe and ascribe value to respondent's activities. We relied on and could not objectively verify respondent reported activities and despite attempts to limit them, we cannot preclude socially desirable responses. While we interviewed almost all (96%) relevant managers in the selected districts for our study, this nonetheless resulted in a small sample size, that is, the study lacked sufficient power to develop a more comprehensive multivariate model. We also had to group together health service and information managers. Choosing one district and sub-district in two provinces out of nine in South Africa limits generalisation to other provinces.

### A ‘hybrid’ indirect M&E programme with horizontal production and vertical use of HIV data

Our study reveals several key findings. First, horizontal and vertical managers exercise similar degrees of authority over the HIV M&E function overall, though horizontal managers exercise more authority over HIV information production, while the use of HIV information is largely under the control of vertical managers. Second, HIV managers largely function outside the district management structure and use HIV data in even smaller sub-programme specific silos, usually excluding horizontal managers. This represents a more extreme form of verticalisation than the usual models in the literature. In considering these two findings in conjunction with our previous work which showed that the HIV M&E system is predominantly operationally integrated with the DHIS (24), we find that the HIV programme M&E is partly operationally and partly administratively integrated within the health system M&E function. This concurs with the discourse regarding a ‘false dichotomy’ between horizontal and vertical approaches (31), and observations that programmes tend to lie along a continuum from integrated to fully vertical (32). According to Unger et al. (13), disease specific programmes can be: a) *vertical*, that is, no integration; b) fully *integrated*, that is, operational and administrative integration, or; c) *indirect*, that is, operationally integrated but administratively vertical. The HIV M&E system in our study does not fit neatly into any of these categories, but is closest to the indirect model (as it is largely operationally integrated and largely administratively vertical). We therefore refer to it as a *hybrid indirect* programme.

Third, despite the top-heavy HIV management structure at provincial level, vertical and horizontal managers play similar roles, mostly performing the same HIV M&E tasks but only on discrete sub-sets of the HIV dataset. This suggests duplication, overlapping roles and thus inefficiency. It needs to be established whether this is because of a lack of clarity regarding division of roles. Related research on decentralisation shows poor role clarity limits managers’ ability to exercise their delegated management authority (19, 33). Finally, vertical managers possess higher HIV M&E knowledge scores (70% vs. 51%) and HIV data use scores (70% vs. 48%) than horizontal managers. The gap between these manager groups indicates that investment is required to support horizontal managers’ capacity to use data. Or alternatively it could describe a particular clearly defined role that some HIV programme managers could play as technical experts and still leave space to deploy some HIV programme managers to other roles in particular in resource-constrained environments. Conversely horizontal managers exercise higher degrees of authority over HIV data collection and collation domains. This could be because they systematically over-estimated their practice or more likely because good knowledge of HIV indicators may not be essential for coordinating these domains.

### Horizontal production and vertical use of HIV data: drivers and health system implications

Funding arrangements in health systems are context-specific and have been found to be determinants of integration (30). This may drive the vertical use of HIV data in our study as most HIV funding is given through a conditional grant which is different to regular funding that comes through the provincial health budget (20). There are also views that because of DHIS weaknesses and horizontal managers’ limited knowledge of disease control issues, vertical M&E systems should be maintained (24) and vertical programmes retained under the control of vertical managers (19). Such attitudes may perpetuate vertical managers’ dominance in using HIV data rather than improving the capacity of horizontal managers. In South Africa, conditions attached to the HIV conditional grant require HIV managers to submit a separate report on HIV expenditure (20). In this way, vertical managers are forced to monitor nationally defined HIV programme indicators. In contrast, this is not required of horizontal managers who have wide ranging and poorly defined performance measures that in many instances ignore data and health outcomes (34).

An M&E system characterised by horizontal production and vertical use of programme information perpetuates the idea that horizontal managers should merely produce and submit programme data ‘upwards’. This contradicts DHS strengthening ideals, which are about improving horizontal managers’ capacity to use information for management. As information use is the ultimate purpose of M&E (35), it is not beneficial for horizontal managers to play a key role in information production and then not use it optimally. In settings pursuing DHS strengthening or decentralisation, managerial reforms to ensure horizontal managers are the primary users of health (including disease-specific) information are imperative.

A well-functioning DHS is however a necessary foundation if district managers are to assume authority over programmes. That a functional DHS is not fully established in South Africa (34) could hinder administrative integration. Similarly, a functioning middle management is important for successful administrative integration (13), as poor managerial capacity can limit the extent to which managers exercise authority over newly allocated roles (36). Arguing that horizontal managers should not manage programmes because of limited capacity is circular and simply reinforces their incapacity. It may discourage agency in managers who should be leading the development of their districts. Rather, investments to equip them with technical skills are imperative.

### Recommendations

In the absence of conclusive evidence on the most effective integration models (37), countries are advised to adopt context-specific arrangements that optimise health system benefits (8). In this section, we draw on our findings to propose actions for South African policy-makers. Our case study findings are unlikely to be unique to the HIV programme. We anticipate broader relevance for other disease-specific programmes in South Africa which are managed by parallel bureaucracies, and particularly those with dedicated M&E systems.

In policy contexts like South Africa where DHS strengthening is a priority, the continuing role of vertical programme management structures warrants revision as silo structures could undermine DHS development. However, as we have shown, programme models can be quite complex and any decisions to revise existing arrangements, for example, by shifting authority from vertical to horizontal managers or integrating governance structures, need to consider the context. If integration is to be pursued in a weak health system, a phased incremental process, while building horizontal management capacity, is advised to avoid undermining local absorptive capacity (2, 8). In the interim, decision-makers could ensure that horizontal and vertical managers engage in dialogue and joint planning and monitoring (30). Linked to this should be clarity in allocation and application of their respective roles, as ignoring this can exacerbate confusion about how vertical managers should engage with horizontal management structures (18). Clarity regarding district managers’ delegated authority over vertical programme functions is also important.

Finally, we recommend further research to evaluate integration of other disease-specific programmes with the M&E as well as other health system functions and to also understand whether and how clarification of roles, individual management capacity, and health system funding models influence administrative integration. Considering our methodological limitations, we recommend further research to test our scales on larger samples using more precise definitions of management capacity, in order to produce more robust measurement scales. That programmes rarely fit within prevailing conceptualisations of ‘integrated’ or ‘vertical’ and that formal delegated authority may vary across settings adds complexity in that measurement tools may have to be quite context-specific to capture nuances. This may limit applicability in cross-country studies.

## Conclusion

In light of the increasing focus on health system strengthening and integration, our research makes a contribution by providing a method and scales for measuring and monitoring administrative integration. We anticipate that these will be strengthened further by empirical testing on larger samples and varied settings. In applying this method to South Africa's public sector HIV programme, we find that HIV M&E coordination is generally not administratively integrated, characterised by horizontal managers exercising little authority in using HIV data, and vertical managers using HIV data in sub-programme silos. We argue that this programme model should not be sustained as it potentially undermines aims of integrated district health management.
